# The externalities of energy production in the context of development of clean energy generation

**DOI:** 10.1007/s11356-020-07625-7

**Published:** 2020-02-27

**Authors:** Andrzej Bielecki, Sebastian Ernst, Wioletta Skrodzka, Igor Wojnicki

**Affiliations:** 1 Department of Applied Computer Science, Faculty of Electrical Engineering, Automatics, Computer Science and Biomedical Engineering, AGH University of Science and Technology, Al. Mickiewicza 30, 30-059 Kraków, Poland; 2 Department of Statistics and Econometrics, Faculty of Management, Częstochowa University of Technology, Armii Krajowej 19B, 42-200 Częstochowa, Poland

**Keywords:** Renewable energy, Electricity generation, External costs, Human health impact, Environmental impact, Risk

## Abstract

In this paper, we present a comparative review of the externalities of electricity production. First of all, the environmental impact is considered. A discussion of the influence of various electricity production processes on human health follows. The studies are conducted in the context of historical development. Current trends, as well as a historical background that resulted in the changes that can be observed today, are presented. The considerations are supported by a few case studies. Analysis of perspectives for the development of electricity generation methods, in particular the indication of clean energy sources and the perspectives of their exploitation, is the main aim of this paper.

## Introduction

The power industry is a branch of economics that has been developed since the nineteenth century. This development has its own dynamics manifesting, among others, in a deep restructuring that consists in changing the percentage participation of various energy sources. In the beginning, it was based only on coal combustion. Subsequently, other combustion methods were introduced such as natural gas and oil. The introduction of renewables was a significant game-changer. Historically, since the last quarter of the nineteenth century, especially in the USA, hydroelectricity contributed to a great extent in electric energy generation (Sternberg 2008). Since the second half of the twentieth century, many renewables have been introduced: wind, solar, and biomass. Moreover, after the Second World War, nuclear energy became a promising alternative. Each of these energy sources has its own characteristics in terms of technology, economy, and influence on the natural environment. The last one concerns both regular electricity production and consequences of malfunctions. More than 150 years of power industry development make broad analyses of the impact of electricity generation on the natural environment possible.

The power industry generates a broad spectrum of problems related to the protection of the environment. It should be considered not only in the context of its influence on nature but also through the prism of the effects on the quality of human life, including health and man-made infrastructure. Moreover, these aspects of the power industry are regulated by the laws specific to various regions of the world. In general, renewable energy and non-renewable resources have their own specificity in the aforementioned context.

The studies of the externalities of electricity generation are the main aim of this paper, while environmental aspects are the main topics of the studies. Some other externalities, however, are also taken into consideration. The studies are conducted from a wide historical perspective. Not only years of research covering the second half of the twentieth century were taken into account but also some earlier ones, even dating back to the nineteenth century. Analysis of electricity generation methods, in particular, identification of clean energy sources and perspectives for their development, is the authors’ primary intention.

The paper is organized in the following way. In the “Characteristics of power generation technologies” section, the characteristics of all common power generation technologies are presented. The “Holistic aspects of power industry economics” section discusses the relations between power generation and environmental protection in a broad sense, including economic aspects. The following section concerns several case studies, which are discussed in detail. A short summary is given at the end of the paper.

## Characteristics of power generation technologies

Electric energy, as it occurs in nature, cannot be directly used to fulfill man’s needs, especially on an industrial scale. Therefore, it needs to be generated in power plants by means of utilizing other types of energy. These plants can be divided, with respect to the method used to generate electricity, into the following categories.

### Nuclear power plants

where electricity is produced in the process of nuclear fission. The currently used technology utilizes the heat resulting from a nuclear reaction; therefore, nuclear power plants belong to the group of thermal generators. On the one hand, nuclear plants have unquestionable advantages: the process generates practically no environmental pollution—the radiation produced during nuclear reactions can be efficiently screened—failures occur rarely, and the estimated reserve of uranium should cover the energy needs of mankind for several hundred years. On the other hand, failures, although rare, can be very dangerous. So far, only two breakdowns—in Chernobyl (1986) and Fukushima (2011)—were classified as major accidents, i.e., they were assigned the highest, seventh level according to the International Nuclear Event Scale (INES), developed collaboratively by the International Atomic Energy Agency (IAEA) and the Nuclear Energy Agency, a part of the Organization for Economic Co-operation and Development. Nevertheless, the effects of such accidents are directly noticeable in large areas, and they have indirect global influence. Radioactive waste is another major disadvantage. Its storage and management are among the biggest problems related to nuclear energy. The aforementioned problems lead to a decrease in nuclear power generation: Germany adopted a regulation concerning the gradual decommissioning of nuclear power plants in 2002, and many more countries display similar trends. It should be mentioned, however, that since 2012, global nuclear power generation has been increasing, although it is still lower than it was 10 to 15 years ago.

### Conventional thermal power plants

where thermal energy is obtained through chemical combustion of fossil fuels: coal, crude oil, or natural gas. These plants, especially the coal-based ones, cause significant contamination of air, water, and soil by emitting toxic substances and carbon dioxide (Air pollution from electricity-generating large combustion plants 2008; Kucowski et al. 1997). ExternE^1^ provides emission comparison which is presented in Table 1. In 2008, the European Environment Agency published a report documenting the emission of pollution produced by conventional power plants within the EU (Air pollution from electricity-generating large combustion plants 2008). In Poland, most electricity is produced in coal-based plants, along with all the consequences: Greenpeace Poland indicates that emissions by Polish coal-based power and heating plants cause almost 5400 premature deaths each year (Myllyvirta et al. 2013). In contrast, the combustion of natural gas is a relatively clean technology. Recent research indicates that some gas combustion technologies could reduce health impact costs (AlRafea et al. 2016). It should be mentioned, however, that many countries (including Poland) do not have the necessary reserve for that fuel to constitute the basis of their power production. Russia is a major supplier of gas for Europe, but it is rather risky to have national energy dependent on the Russian supply as the country uses gas provision limitations to exert political pressure (Stulberg 2015).

**Table 1:** Typical emissions by coal and gas power plants in the EU, in g/kWH_*e*_ (Rabl and Spadaro 2016; Samadi 2017; Hansen 2019)

	Hard coal, condensing power plant			Natural gas combined cycle		
	Upstream	Plant	Total	Upstream	Plant	Total
CO_2_	30.09	730.2	761.1	46.9	355.5	402.4
CH_4_	2.2	0.0	2.2	1.0	0.0	1.0
CO$_{2eq}^{a}$	84.8	730.6	815.4	71.6	355.6	427.3
PM_2.5_	0.01	0.00	0.01	0.00	0.00	0.01
PM_*co*_	0.02	0.03	0.04	0.00	0.00	0.00
NOx	0.33	0.55	0.88	0.15	0.27	0.42
SO_2_	0.24	0.55	0.79	0.14	0.01	0.15

Mining causes deep soil destruction. In the process of mining of natural resources, original soils become lost or buried by wastes (Bradshaw 1997). This is caused by deep coal mining operations and coal processing. In particular, mine spoil heaps are not pleasant milieus for plant vegetation because they are composed of coarse rocks. Furthermore, such spoils are characterized by unfavorable pH, low organic matter content, drought arising from the coarse texture, and oxygen deficiency. The other factors, such as poor water holding capacity, acidity, salinity, low level of nutrients, and high rate of erosion, also limit plant vegetation (Hazarika et al. 2006). All of the specified factors constitute environments that are suitable for neither plant nor microbial growth (Jha and Singh 1991; Dutta and Agrawal 2002).

On the other hand, however, it has been demonstrated that the processes of natural succession can reconstruct fully functioning soils. Some species of plants can fix and accumulate minerals rapidly and in sufficient quantities to enrich the possibility of vegetation of numerous other species (Bradshaw 1997; Stefanowicz et al. 2015). Sometimes, however, it is necessary to artificially introduce the plant species that are most appropriate for the restoration process. In the mined lands, the extreme soil conditions mentioned above can occur, preventing plant growth. It is crucial to identify and remove them. Otherwise, the restoration process may be delayed significantly or can even fail after a few years (Bradshaw 1997). To rehabilitate post-mining regions successfully and aid it as effectively as possible, a proper restoration activity should take place. What makes it more important is the fact that, usually, natural revegetation of the coal spoils is sparse and patchy with low plant cover, especially at the initial stage of regeneration (Baig 1992). In particular, due to the unfavorable post-mining environments, the absence of plants on fresh coal-mine soil can be observed (Banerjee et al. 1996; Ekka and Behera 2011). Thus, at the early stage of rehabilitation, a greater contribution of grass species is recommended, whereas a greater diversity of non-grass plant species can be observed with the increasing age of soil (Ekka and Behera 2011). Since efficient rehabilitation of post-mining regions depends on numerous factors, there is a great need for computer decision support systems (Bielecka and Król-Korczak 2010a, b). Nevertheless, the aforementioned disadvantages lead many countries to invest in renewable energy sources.

Conventional thermal power plants deplete natural resources. The quantitative estimation of these resources is given in Table 2. The actual numbers fluctuate in different years. This is caused by the consumption of the resources, as well as technological advancements of survey and mining methods. Even though the 2018 estimation of the world’s coal reserves is much higher than the one from 1974, Bp Statistical Review of World Energy (2018) indicates that its current consumption can only be satisfied for just over 130 years. Natural gas and oil consumption can be satisfied for a period that is three times shorter. It should be noticed that these estimates can increase because of technological advances. It should be stressed, however, that there is a consensus among the scientific community that the risk posed by climate change causes the necessity to cut the use of fossil fuels.

**Table 2:** Estimates of accessible world’s natural resources

	1974^*a*^	1986^*b*^	1995^*c*^	2010^*d*^	2010^*e*^	2018^*f*^
Coal [mld Mg]	476.0	566.0	716.5	405	411.3	718.3
Lignite [mld Mg]	219.0	272.0	315.0	456	414.7	316.7
Petroleum [mld Mg]	89.7	94.0	140.6	163.0	181.7	239.3
Natural gas [bilion m^3^]	64.8	85.5	141.3	185.5	187.5	193.5

^*a*^Survey of Energy Resources, 9 World Energy Conference. WEC, Detroit 1974. ^*b*^Survey of Energy Resources, 13 World Energy Congress. WEC, Cannes 1986. ^*c*^Survey of Energy Resources, 16 World Energy Congress. WEC, Tokyo 1995. ^*d*^Survey of Energy Resources, 21 World Energy Congress. WEC, Montreal 2010. ^*e*^BP Statistical Review of World Energy 2010. ^*f*^BP Statistical Review of World Energy 2018

### Water power plants

which generate electricity either by using the kinetic energy of flowing water or by the potential energy of water gathered in dams in the Earth’s gravitational field. In Poland, it is impossible to utilize the kinetic energy of tides, as they are practically absent in the Baltic sea. However, natural conditions make the construction of small flow plants (i.e., plants that do not require dams) on rivers possible. Dams are the only large water plants used in Poland that until recently were classified as a clean source of energy. Nevertheless, recent research shows that they cause intensive emission of methane from organic remains that are accumulated in the standing water of the reservoir (Trojanowska et al. 2009; DelSontro et al. 2010). Furthermore, the presence of a large dam changes the ecosystem significantly: completely different organisms develop if a large container appears in place of a swift river. Moreover, a large reservoir greatly increases evaporation, which alters the air humidity in a large area. Water aeration decreases and the bottom of the reservoir is silted.

### Power plants based on the combustion of biomass

or its decomposition products. According to a common conviction, the combustion of biomass is more environmentally friendly than the combustion of fossil fuels; however, that fact is not reflected in the results of scientific research. First of all, the production of biomass on an industrial scale leads to expansion of agricultural areas or to the change of use of those already existing—they are shifted from production of food and animal feed to cultivation of energy crops. This not only leads to the increase of food and feed prices but also causes the degradation of the environment, e.g., to the consumption of water resources and decrease of biodiversity (Armaroli and Balzani 2007; Naylor et al. 2007). Moreover, the combustion of biomass produces specific harmful substances that result from the combustion of protein and fat. The calorific value of biomass is several times lower than that of coal. Therefore, the volume used to produce a certain amount of electricity is much higher. As the EU member states are obliged to obtain a certain part of their energy from renewable fuels, Poland, in an effort to decrease the implementation costs, started adding biomass to the coal used in conventional plants. This not only caused technical problems, such as corrosion of boilers, varying boiler load or mill, and ventilator failures, but also led to series of tragic breakdowns, such as fires and explosions occurring from 2005 to 2012, which resulted in casualties (Chojnacki 2012).

### Geothermal power plants

use the Earth’s thermal energy. The energy is harvested from high-temperature resources located deep underground. The heat is carried back to the surface by fluid circulation through hot springs or heated rocks. Potential risks concern groundwater pollution and releasing hydrogen sulfide and other gases to the atmosphere. Fortunately, using natural geysers is risk-free. To use such geothermal energy to generate electricity, the hot spring temperature has to exceed 150^∘^C, it cannot be deeper than 3000 m, and it has to be potable water. There are no such hot springs in Poland. However, in Iceland, in 2010, 26% of overall electricity generation came from such springs (Kaczmarczyk 2011). It increased to 27% in 2013 (Ragnarsson 2015), and 29% in 2014 (Shortall and Kharrazi 2017; Saeorsdottir and Saarinen 2016). In the Philippines, the contribution was 11% in 2013 (Balangue-Tarriela and Mendoza 2015), 14% in 2014 (Bertani 2015), and there is no further growth (Mondal et al. 2018). The above data show that countries that have the hydro-geological potential of geothermal energy exploitation for electricity generation might be able to obtain its significant share in the total production. It should also be stressed that there are countries that have huge geothermal potential that has not been utilized so far. Indonesia, which is situated in the ring of fire in volcano line and therefore has optimal conditions for harvesting geothermal energy, generates less than 5% energy from such sources. The government, however, has a plan for dynamic development of geothermal power plants (Pambudi 2018).

### Solar power plants

harvest solar energy. The electricity can be obtained through a direct process by using photovoltaics (PV) or through an indirect one by harvesting heat and transforming it into electricity (Tytko 2009). Statistical predictability is a significant advantage of such power plants—particular geographic region insolation is a well-known factor. Unfortunately, there are not too many locations where energy could be harvested in such a way, taking into account the economic and technological issues due to insufficient insolation, which is a serious disadvantage. The solar energy in Poland is used harvested only locally and mostly for heating. PV used to be underestimated and criticized (Zapałowicz 2010). However, due to ongoing efficiency improvement, it has already proved to be a viable technology. For instance, it is reported that both the unit cost of photovoltaic cells and the cost of solar electricity production decrease (Ferreira et al. 2018). Moreover, PV can be located on existing infrastructure, such as rooftops and facades. It reduces the problem of area consumption, at least for distributed generation. It has to be pointed out that harvesting solar energy does not generate any byproducts as it constitutes a truly clean energy source. That is why there is a lot of research directed at improving the harvesting efficiency and new materials (Schwarzbözl et al. 2006; Polman et al. 2016). Combining electricity and heat generation in a hybrid system is one of the examples (Szymański 2011). It should be noted that photovoltaics is not developed to an extent that would make us aware of all the problems associated with the full cycle of its use. Nevertheless, the problems with the storage of materials from used solar panels have already been identified. However, there are indications that second-generation PV panels can be effectively recycled (Savvilotidou et al. 2017). Furthermore, a cradle-to-grave life cycle assessment of perovskite solar modules (the third generation of photovoltaics) showed that they have a very short energy payback time and this technology is potentially the most environmentally sustainable (Gong et al. 2015). Nevertheless, according to the aforementioned poor recognition of the problem of waste treatment, this problem should be monitored and studied intensively.

### Wind power plants

transform the kinetic energy of wind into electricity. Harvesting wind energy, similarly to solar power plants, does not generate any byproducts—it is ecologically clean. Furthermore, exploiting wind energy is far less limited than solar energy. Even though solar energy harvesting is available across the globe in a foreseeable 24-h cycle, the energy production based on it is more limited than the one based on wind energy in the frigid and north temperate zones, taking the limitations of the current energy harvesting technologies into account. It should be stressed, however, that because of intensive research in this domain (Sesto and Casale 1998) and advancements in energy harvesting, it could change in the near future. Windmill technologies are also prone to malfunctions. Nevertheless, it has to be pointed out that wind-based energy generation is the least predictable which is a significant drawback. Development of such energy harvesting requires appropriate tools for assessing demand (Bęk and Bielecki 2007; Dęsal et al. 2010) and precise wind forecasts (Callaway 2010; Damousis et al. 2004; Fan et al. 2009; Lei et al. 2009; Taylor et al. 2009) which leads increased to generation predictability (Focken et al. 2002; Landberg 1999; Lei et al. 2009; Watson et al. 2010).

To sum up, electricity generation based on fossil fuels is the most devastating one for the natural environment. Nuclear generation, while clean, results in challenges of radioactive byproduct storage. Taking a look at the renewable energy sources, it becomes apparent that solar and wind are the only ones that are clean and globally available. The latter offers more economy–wise exploitation possibilities now, which might change due to advancements in energy harvesting technologies (Hirth 2015) in the future. It is reported in literature that regardless of whether the power plant level is taken into consideration or the whole cycle of production is studied, the low-carbon technologies of electricity generation exhibit lower external costs per kWh than the technologies based on fossil fuels (Samadi 2017). It should be also noted that economic calculations that concern the influence of energy production on the environment and human health can only have an auxiliary character because of existing unmeasurable factors. For instance, the value of human life is priceless and “to save an individual in danger no means are spared” (Rabl and Spadaro 2016). In such context, clean energy is all the more desirable. The characteristics of power generation and exploitation technologies are summed up in Table 3. Poland, being the EU member, is required to generate 20% of energy from renewable sources by 2030. It is assumed that it would be obtained mainly from wind and biomass (Holtzer and Holtzer 2012).

**Table 3:** Summary of risks related to different types of power generation

Plant type	Effect on human health	Nuisance for humans	Effect on man-made infrastructure	Natural environment impact	Worst-case disaster scenario
Nuclear	Increased long-term radiation exposure for staff	None	None	Environmental hazards due to nuclear waste storage.	Catastrophic environment pollution and human health hazard; global effects.
Coal thermal	Minor due to modern emission reduction technologies and combustion process optimization. Severe if such technologies are not applied	Depending on emissions	Severe, taking into account mining and distribution.	Depending on emissions for plants; severe regarding mining operations.	Local effects.
Natural gas thermal	Minor	Minor	Minor, including pipelines.	Minor	Local effects.
Water potential	None	Average in case of dams, minor for pumped-storage	Average globally, large locally including transport and communication infrastructure impact.	Average, regionally.	Catastrophic regionally due to floods.
Biomass thermal	Not sufficiently investigated, potentially significant due to organic material combustion	None	None	Significant due to crop fields designated for energetic plants.	Local, in case of bio-gas generator fire.
Geothermal (natural)	None	None	None	None	None
Geothermal (man-made)	Groundwater pollution, air pollution	Groundwater pollution, air pollution	None	Groundwater pollution, air pollution	Groundwater pollution, air pollution
Solar concentrated	None	Large area needed	None	Lethal threat to flyby birds	Fire, releasing steam to atmosphere
Solar PV	None	None	None	Area consuming, unresearched potential problems due to PV recycling.	None
Wind turbines	None	Noise, vibration, flashing glare, large area.	May affect propagation of RF signal.	Animals: noise, vibration; Avians: collision risks.	None

## Holistic aspects of power industry economics

The common way of economic assessment of electricity delivery concerns its generation and distribution. The power industry, however, globally affects its whole environment, including human health. These aspects should be taken into consideration while assessing the total costs of electricity generation. It should be stressed that the impact on the environment and on human health, not the actual cost of electricity, is analyzed here. The costs are used only to estimate this impact and, as such, they have an auxiliary character.

### Costs of electricity production

In recent years, the ongoing technological changes in the electricity market, as well as the new geopolitical situation, have contributed to the reduction of the price of energy raw materials, crude oil in particular. However, lower prices can divert the focus from the World’s basic energy problems—energy security, competitiveness, and climate change. Efficient use of energy resources plays a crucial role in the economic growth and sustainable development of societies. The World Commission on Environment and Development (WCED) emphasizes the significance of long-term exploitation perspective of accessible resources for the improvement of human welfare (WCED Bruntland Commission 1987). The concept of sustainable development in relation to the electricity sector takes the absorption capabilities of the environment and the reduction of negative ecological and climate consequences into consideration. Comparison and assessment of electricity generation technologies, supply chains, and estimated costs of electricity production should be based on comparable measures, which tackle the use of resources as well as health and environmental hazards. A social opportunity cost encompasses the society’s outlays and the costs connected with using and processing energy resources and encountering unfavorable environment changes as well as degraded human welfare (Zhen et al. 2018). It embraces both direct costs and external costs in the entire energy transformation chain. In general, overall costs of electricity consists of three main components—costs associated with energy production, the costs related to the distribution grid, and external costs, see Nuclear Energy Agency (2019), pages 14–22. The first component includes the cost of building and operating a power plant. The second one includes, first of all, the costs of extending, reinforcing, or connecting to the system as well as the costs of maintaining reserves and additional dispatchable capacity. The above two components constitute the said direct costs. The third one, external costs, includes the impact on both communities outside the electricity sector and the environment. They usually are not included in the price of energy, but the whole society bears them as a result of energy production in a given way. Human health damage, caused by the emission of pollutants during combustion processes in thermal power plants, is an example of an external cost. In general, the health effects of energy sources are regarded as one of the main types of external costs. Climate and environmental changes, first of all pollution, are also significant ones. This paper is focused on the environmental changes, but health damage is also considered.

Life Cycle Assessment covers the search for resources, extraction, processing, transport, processing and disposal of waste, and costs related to environmental impact (Li et al. 2017; Lohse 2018). It also focuses on the costs related to the implementation of infrastructure and the demand for electricity at the initial and final stages. A proper, detailed assessment of all stages of a fuel cycle is the prerequisite of a correct comparison of options of electricity production, with sustainable development in mind. The selection of the primary electricity carrier should be based on the estimation of the social costs necessary to acquire and process it (e.g., combustion or the fission chain reaction), with the subsequent consideration of all consequences.

The globally increasing cost competitiveness of technologies in the field of renewable energy is most visible in the Gulf Cooperation Council countries. Renewable energy technologies, such as photovoltaic cells and CSP, are increasingly competitive compared with other sources. The global weighted average cost of electricity for photovoltaic projects, ranging from 6 to 10 US cents/kWh, decreased in 2017 by 73% compared with 2010. The electricity production price of 5.84 cents/kWh in 200 MW Phase II of Dubai’s Mohammed bin Rashid was comparable with the price of electricity generated from crude oil at the price of 20 USD per barrel. In the same period, the weighted average cost of electricity in onshore wind farms, oscillating around 4 cents/kWh in areas with strong winds, dropped by 22% (IRENA, 2018).

### External costs

The estimation of external costs of energy-related investments is a complex process. To analyze them in detail, it is necessary to precisely estimate the cost of every damage caused by individual pollutants and a potential catastrophe in the whole fuel cycle. Nevertheless, some estimates are impossible or they do not have enough precision (van den Bergh and Botzen 2015). The lack of international standards concerning some costs of damages hinders analysis and makes it impossible to compare the results.

Literature defines the external costs of electricity generation technologies by focusing mainly on the negative effects of the technical processes related to the production of electrical energy and heat. These include construction and liquidation of power plants, extraction and transportation of resources, and emission of pollution (Friedrich and Voss 1993; Longo et al. 2008). Comparison of the environmental impact of different electricity production methods is usually based on the analysis of external costs (Georgakellos 2010; Krewitt 2002; Corona et al. 2016). When it comes to renewable energy sources, the external costs are relatively low, especially in comparison with the conventional energy production methods which are the main source of greenhouse gas emission (GHG). That, in turn, leads to health problems, decreased biodiversity, loss of crops, degradation of building facades, and corrosion of materials (Mirasgedis et al. 2000; Máca et al. 2012; Bridges et al. 2015; Streimikiene and Alisauskaite-Seskiene 2014). The works by Rowe et al. (Empire State Electric Energy Research Corporation 1996) and Lee et al. (1995), also called the RFF/ORNL research^2^, are recognized as the first comprehensive elaborations on the external costs in a fuel cycle. They are focused on the entire fuel cycle of different kinds of power plants. Another subsequent analysis of external costs connected with the emission of pollution, catastrophes, and radiation caused by English coal, gas, and nuclear power plants was carried out by Pearce (1994). In 1997, Bhattacharvya (1997) analyzed the costs of a hypothetical Indian power plant of the capacity of 210 MW, based on coal. The values of life and disease costs were based on the data from the literature. In 1999, Krewitt et al. (1999) used the bottom-up method to determine the average external costs connected with the generation of energy in the mines in Germany and Europe. The authors used the EcoSense model^3^ which comes from the ExternE methodology, as well as the CORINAR database (Core Inventory Air Emissions).^4^ An interesting analysis of the research methodology with regard to the estimation of external costs was presented in 2002–2004 by Sundqvist and Söderholm (Sundqvist and Soederholm 2002; Söderholm and Sundqvist 2003) (see Table 6 for details). They discovered that the analyzed research process was biased because it was carried out in relation to a specific pollution source in a specific place. The estimations of external costs differ significantly in the research concerning Europe and developing countries. While comparing the results of American research on different electricity generation technologies, Sundqvist (Sundqvist and Soederholm 2002) noticed significant diversification of the monetary value of the external costs connected with both the same and different energy generation technologies (Table 4).

**Table 4:** Statistical analysis of external costs for various energy technologies [US cent/kWh] (Sundqvist and Soederholm 2002)

	Coal	Oil	Gas	Nuclear	Hydro	Wind	Solar PV	Biomass
Min	0.06	0.03	0.003	0.0003	0.02	0	0	0
Max	72.42	39.93	13.22	64.45	26.26	0.80	1.69	22.09
Average	14.87	13.57	5.02	8.63	3.84	0.29	0.69	5.20
Median	8.30	11.62	3.80	1.03	0.32	0.32	0.63	2.86
Standard deviation	16.89	12.51	4.73	18.62	8.40	0.20	0.57	6.11
Number of observations	29	15	24	16	11	14	7	16

Sundqvist also used variance analysis to determine the influence of the method, type of fuel, inclusion or exclusion of the whole fuel cycle, income, and population density on the final estimation of external costs. Spalding-Fecher and Matibe (2003) carried out research on the external costs of electricity production in the Republic of South Africa. The harm caused by air pollution was adjusted to the population growth, emission changes, and inflation. Roth and Ambs (2004) performed a complete analysis of the costs of electricity for 14 production technologies in a hypothetical power plant in the North-East of the USA. The analysis encompassed the damages caused by air pollution, energy safety, electricity transmission and distribution costs, and other environmental threats such as the change of land allocation or the pollution of groundwater. Maddox et al. (2004) analyzed the fuel cycle of all coal generators in New South Wales in Australia in order to determine the total emissions of PM_10_, SO_2_, NO_x_, and CO_2_.

One of the most comprehensive attempts at the estimation of external costs in the power industry was performed as a series of European projects between the early 1990s and 2005 under a common name, ExternE. As a result, the research brought a methodology to analyze the ways in which pollution affects the society (Bickel and Friedrich 2005). The exposure response was determined at the EU scale. The health effects of air pollution and their monetary values were defined. They included elements such as the value of statistical life time reduction, malfunctions in the entire power generation cycle, and greenhouse effect. The methodology was also used to support the decision-making process in energy policy and environmental protection. There were areas, however, such as determination of the monetary value of the results of mortality, soil acidification, or eutrophication, which needed further analysis. After 2005, there were many other projects such as NewExt,^5^ ExternE-POL,^6^ NEEDS,^7^ and CASES^8^ which were aimed at developing the methodology, so that it could cover even broader range of unfavorable external effects caused at the stage of production, transportation, and utilization of energy fuels. The EcoSense computer software was used to value the health and environmental harm accompanying the production of electricity.^9^

Rafaj and Kypreos (2007) used the global MARKAL model^10^ to analyze the influence of the internalization of external costs of electricity production^11^. The countries were grouped into five regions: two regions with the OECD countries, one region with transitional countries, and two regions with developing countries. Likewise, in 2007, Klaassen and Riahi (2007) examined the approach in which all the environmental harm was internalized. The data sets and the model framework used in the elaboration came from the following programs: Scenario Generator (SG),^12^ MESSAGE^13^, and the CO2DB technologies database.^14^Biegler et al. (2009) estimated the external costs of coal and gas power plants in Australia. In order to estimate health harm, he examined the emissions of CO_2_, PM_10_, SO_2_, and NO_x_. An interesting analysis was carried out by the National Research Council in 2010 (Hafemeister et al. 2011). The authors calculated the external costs caused by the emission of pollution by 406 existing coal power plants and 498 existing gas plants. The results obtained by the NRC were compared with the results of RFN/ORNL and ExternE (Table 5).

**Table 5:** Summary of estimates from external cost studies, in millions per kWh (expressed in 2010 US dollars) (Burtraw et al. 2012)

Source	Coal	Peat	Oil	Gas	Nuclear	Biomass	Hydro	PV	Wind
RFN/ORNL	2.3	−	0.35–2.11	0.35	0.53	3	−	−	0
ExternE	27–202	27–67	40.3–148	13.4–53.8	3.4–9.4	0–67	0–13	8.1	0–3.4
NRC	2–126	−	−	0.01–5.78	−	−	−	−	−

A mill is one-tenth of a cent or one-thousandth of a dollar; PV is photo voltaic

Significant disparities can be found in the presented results due to the differences in methodology, place, population, and the research scope—parameter assumptions and technical specifications. Dimitrijević et al. (2011) estimated the external costs of coal-based heat and power plants in Bosnia and Herzegovina. Burtraw and Krupnick (2012) surveyed the methodologies for the estimation of real electricity costs for the energy technologies available in the USA. The research was contracted by the Renewable Energy Policy Network for the 21st Century. Mahapatra et al. (2012) analyzed the environmental impact of coal combustion in twin cities Ahmedabad and Gandhinagar in West India. Similarly, Nkambule and Blignaut (2012) estimated external costs of the transportation of coal to the power plant in South Africa. Castelo Branco et al. (2013) examined the life cycle of coal power plants in Brazil. In their analysis, the authors focused solely on capturing and storing carbon dioxide (CCS), disregarding other pollutants. Brandt et al. (2013) developed the integrated modeling system EVA (economic valuation of air pollution) based on a chain of impact pathways. Identifying anthropogenic sources of human health deterioration in Europe and Denmark was the main goal of their work. In 2014, the external costs of electricity production in Lithuania were estimated by Streimikiene and Alisauskaite-Seskiene (2014) who applied the ExterE methodology. Rentizelas and Georgakellos (2014) used the life cycle of external costs in order to optimize the process of electrical energy production. Borozan et al. (2015) examined and estimated the internalized costs of a heat and power plant in Croatia. In order to determine the social costs connected with the production of energy, the impact pathway method from the ExternE methodology was applied.

Corona et al. (2016) examined the use of Full Environmental Life Cycle Costing (FeLCC) methodology to evaluate the economic performance of a 50-MW parabolic trough Concentrated Solar Power (CSP) plant operating in hybrid mode with different natural gas inputs (between 0 and 30%). The analysis incorporated the estimation of external costs associated with atmospheric emissions on six categories: Human Health, Loss of Biodiversity, Local and Global Damage to Crops, Damage to Materials, and Climate Change.

Samadi (2017) categorized the relevant types of costs, differentiating between plant-level, system, and external costs as the main categories. He discussed the relevance of each type of cost for each generation technology. Rečka and Ščasný (2017) estimated the external costs connected with the production of electricity in the Czech Republic.

A recent analysis for Lithuania (Streimikiene and Alisauskaite-Seskiene 2014) has shown that the future energy policy should be oriented towards renewable energy because the external cost for this type of energy is significantly lower than for conventional sources. The analysis took diverse external cost categories, electricity generation technologies, life cycle stages, and the 2010–2030 time frame into account.

The literature on the subject lists the following research areas of analysis of external costs of electricity generation technologies:

- Social costs of GHG emissions (minus market costs of GHG emissions) (van den Bergh and Botzen 2015; Zhen et al. 2018),
- Impacts of non-GHG pollution (European union emission inventory report 1990-2016 (Tech. Rep.) 2018),
- Landscape and noise impacts (Droes and Koster 2014),
- Impacts on ecosystems and biodiversity (beyond those related to climate change) (Walston et al. 2016),
- External costs associated with radionuclide emissions (Burgherr and Hirschberg 2014).

The literature distinguishes two approaches in the process of external cost estimation: the abatement costs approach method and the damage costs approach method. The first one, based on the use of the size of control activities or those that eliminate damage, is the measure of avoided external costs. The value of external costs estimated according to this method is usually overstated. The damage cost approach method uses the direct measurement of the actual external costs in the valuation process. It can be done in two ways. Using the top-down approach, on the basis of aggregated data, the external cost indicators for the entire national economy are estimated based on the total emissions of a given type of pollution. It estimates the results of the costs of specific emission sources (Table 6).

**Table 6:** External costs of different energy technologies [US cent/kWh] (Sundqvist and Soederholm 2002)

Study	Country	Source	External costs
ORNL&RtP (1994)	USA	Coal	0.11–0.48
		Oil	0.04–0.32
		Gas	0.01–0.03
		Nuclear	0.02–0.12
		Hydro	0.02
		Biomass	0.20
RER (1994)	USA	Oil	0.03–5.81
		Gas	0.003–0.48
EC (1995)	UK/DE	Coal	0.98/2.39
	DE	Oil	3.00
	UK	Gas	0.10
	FR	Nuclear	0.0003–0.01
	NO	Hydro	0,32
	UK	Wind	0.11–0.32
Rowe et al. (1995)	USA	Coal	0.31
		Oil	0.73
		Gas	0.22
		Nuclear	0.01
		Wind	0.001
		Biomass	0.35
van Horen (1996)	ZA	Coal	0.90–5.01
		Nuclear	1.34–4.54
Bhattacharyya (1997)	IN	Coal	1.36
Faaijetal (1998)	NL	Coal	3.84
		Biomass	8.10
EC (1999)	BE, FI, FR, DE, IE, NL, PT, ES, SE, UK	Coal	0.84–72.42
	FR, DE, GR, IT, UK	Oil	2.07–39.93
	AT, BE, DK, FR, DE, GR, IT, NL, NO, PT, ES, UK	Gas	0.26–11.78
	BE, DE, NL	Nuclear	0.02–1.45
	AT, GR, IT, PT, SE	Hydro	0.02–18.54
	DK, DE, GR, NO, ES, UK	Wind	0.05–0.80
	DE	Solar PV	0.05–1.69
	AT, DK, FI, FR, DE, GR, ML, NO, PT, ES, SE, UK	Biomass	0.14–22.09
Maddison (1999)	UK/DE	Coal	0.31–0.71
	DE	Oil	0.78
	UK	Gas	0.13

When it comes to the gaseous pollution resulting from the production of electricity, the external costs are estimated by categories such as human health, material damages, degradation of crops, loss of biodiversity, and climate change. Table 7 presents the contribution of individual pollutants.

**Table 7:** Negative externalities associated with the emissions of air pollutants. Source: European Commission. 2005. Externalities of Energy: Methodology 2005 Update. Edited by P. Bickel and R. Friedrich. Luxembourg: Office for Official Publications of the European Communities

Impact area	Pollutant	Effect
Loss of health	PM, SO_2_, NO_*x*_,O_3_, NH_3_, NMVOC, Cd, As, Ni, Pb, Hg, Cr, dioxins	Premature deaths, cardiovascular diseases, cancer
Material damages	SO_2_, NO_*x*_	Corrosion, degradation of buildings’ elevations
Degradation of crops	SO_2_, NO_*x*_	Decreased productivity of crops, loss in forest stands, changes in the chemical composition of soil
Loss of biodiversity	NH_3_, SO_2_, NO_*x*_, NMVOC	Disturbed ecological equilibrium
Climate change	CO_2_, CH_4_, N_2_O, N, S	Floods, long droughts, losses in farming

SO_2_ is responsible mainly for damage to buildings. Continuous contamination from SO_2_, NO_*x*_, and NMVOC has a negative impact on crops, land, and water ecosystems. The most significant harm to human health is caused by the emission of particulates: SO_2_, NO_*x*_, and NMVOC (Brandt et al. 2013). Elevated levels of particulates affect human health in many negative ways, including increased cancer prevalence (especially lung and breast cancer) (Beeson et al. 1998; Crouse et al. 2010; Demetriou et al. 2012; Dockery et al. 1993; Wei et al. 2012), WHO), increased prevalence of innate lung defects, heart and immunological system anomalies in children (Gauderman et al. 2004; Vrijheid et al. 2011), increased prevalence of asthma or worsening of its symptoms, chronic obstructive pulmonary disorder (Carlsten et al. 2011; Gowers et al. 2012; Delamater et al. 2012; HEI Panel on the Health Effects of Traffic-Related Air Pollution 2010; Trasande and Thurston 2005), higher rates of heart attacks and strokes (Chen et al. 2013; Dominici et al. 2006; Mustafic et al. 2012; Qian et al. 2013; Wellenius et al. 2005; Shah et al. 2013), and higher indexes of neurodevelopmental disorders in prenatal period such as autism spectrum disorder, attention deficit hyperactivity disorder, and reduced intellectual level (Becerra et al. 2013; Chiu et al. 2013; Newman et al. 2013; Perera et al. 2013; Perera et al. 2009; Roberts et al. 2013; Volk et al. 2013; Volk et al. 2011). Pregnant women, children, people suffering from lung diseases, and elderly people are the most vulnerable groups.

The following stages of the analysis of external costs can be distinguished (Kudełko 2012):

1. Emission – determination of the volume pollution emitted by a given source, most often expressed in the units of physical emission per production unit,
2. Dispersion – determination of a change in the measures of the environment quality as the function of emission (e.g., the concentration of emission *g*/*m*^3^),
3. Impact – estimation of the type and size of environmental change (e.g., with reference to human health) with the use of the dose-response functions,
4. Cost – transformation of physical effects into monetary value of external costs (e.g., cost of health loss).

Health effects are measured by the increased mortality of people estimated as premature deaths expressed as the cumulated reduction of life expectancy for the population (Leksell and Rabl 2001). In the ExternE project from 2005, the assumed value of a statistical life was equal to 1 million € (Rabl and Spadaro 2005). The estimated financial value of the reduction of expected YOLL, calculated by a 3% discount rate, is equal to 50,000 € (chronic, long-term hazard) and 75,000 € (short-term hazard) (Watkiss 2005).

The estimation of the monetary value of damages is the final stage of the analysis. The market or book values of lost goods or services are used in order to repair the damage. In the case of buildings, the size of the damage is estimated on the basis of the decrease in their value or representative functions. The value of the latter requires a conditional valuation. The losses in agriculture are determined by the secondary deposition of SO_2_, ozone, and NO_*x*_ by using crop market prices. The determination of health and environmental damages requires a separate methodology. When it comes to the costs connected with human health, either the notion of willingness to pay for health risk or willingness to accept compensation from increased risk is used. When it comes to illness treatment costs, lost remuneration and reduced productivity are also considered. The index defined as the value of statistical life is used, in order to estimate the costs connected with increased mortality. The value of the index is assumed as to be equal to 1–5 million € in case of research conducted in the USA and Europe. The index of the value of a life year, VOLY, is used by ExternE research (Radović 2002). Its estimation uses VSL (value of statistical life) and it is based on the change in the life expectancy connected with the reduced risk of death of five in a thousand people in the next 10 years. The value of the index with a 3% discount rate is equal to 40,000 € (chronic, long-term hazard) and 60,000 € (short-term hazard). The total economic effect of increased mortality, YOLL, is a product of the VOLY index and the cumulated reduction of life expectancy. The final estimations of the costs of damages per one tonne for specific damages and the estimations of mortality and morbidity which pertain not only to health but also the quality of life are considered in the ExternE project. Due to the disproportions in the age structure of populations, income differences, and a few others, there are disproportions in the level of WTP for different regions of the world (Wang et al. 2015). According to the directives of the European Commission, the same monetary values of loss of life or its shortening are assumed for all member states of the EU (European Commission 2003).

The external ecological costs of ecosystems were examined within the framework of ExternE, NEEDS, and CASES projects. External environmental costs are connected with the influence of pollution on crops, damages to materials, and the loss of biological diversity caused by acidification (SO_2_, NO_*x*_, NH_3_), eutrophication (NO_*x*_ and NH_3_), and the use of lands for power plant constructions. There are estimations of the damages caused by the emission in the energy production life cycle, namely ammonia (NH_3_), nonindustrial volatile organic compounds (NMVOC), nitrogen oxide (NO_*x*_), particulates, and sulfur dioxide (SO_2_). The evaluation of the loss of biodiversity is based on the valuation through the willingness to pay (WTP) for maintaining the ecosystem in the initial state. The table presents example results of the measurements of external costs of the emission of classic pollutants within the CASES 2008 project. The values of external costs were obtained through a simulation of some scenarios of emissions reduction in different EU-27 regions. It is assumed that in most cases, except for NO_*x*_, the emission in 2020 will be lower than in 2010. Some external environmental costs have negative values because they have a positive influence on the environment—for example, the NMVOC emission affects biodiversity in a good way, while the emissions of NH_3_ and SO_2_ have a positive influence on crops.

In the case of the nuclear power industry, the examination of external costs should cover the entire fuel cycle. This means that all stages, from the excavation and processing of uranium, transportation of radioactive materials and waste, through the electricity generation itself, and the disposal of radioactive waste to the liquidation of the power plant should be considered. The parameters which should be assumed for the analysis of the external costs that were caused by the nuclear power industry were determined by the NEEDS program, conducted by the European Union (Osán et al. 2009). They are connected with the releasing of radionuclides in the entire fuel cycle and with their influence on human health. In order to examine the external costs that occur in the phase of exploitation and the other parts of the fuel cycle, collective doses caused by globally dispersed radionuclides during the exploitation of a power plant and fuel reprocessing were determined using data on emissions and production of electricity.

Mining of uranium, due to its exploitation, processing, and generation of toxic waste has a significant negative influence on the environment. Contamination of soil and water, including groundwater, is the main problem (Abdelouas 2006). A high concentration of thorium, uranium, and sulfate, as well as low pH, affect the state on both land and aquatic fauna and flora in the neighborhood of the mines. It is reported, for instance, that uranium mining has negative influence on the density of phytoplankton, primarily of the organisms that belong to the Cryptophyceae and Dinophyceae classes (Roque et al. 2009). Phytoplankton is crucial for the efficient functioning of the biological environment as it is the basis of many trophic networks and it is responsible for the production of about 98*%* of the atmospheric oxygen (Roque et al. 2009; Solomon et al. 2014). Therefore, limiting the factors disturbing its dynamics is crucial for maintaining the balance of the biosphere (Solomon et al. 2014). To limit the mentioned factors efficiently, the methods of waste management as well as understanding of their geochemistry are being developed (Robertson et al. 2019).

The cost of greenhouse gas emission and the greenhouse effect are important elements of the total external cost of electricity production. The measurement of the emission of greenhouse gases in the energy production life cycle involves the calculation of the potential for global warming in electrical energy sources. The results are expressed in global warming units, which are an equivalent of carbon dioxide emission (CO_2_e) per unit of energy produced by the source. The whole life of the energy source, from the extraction of materials and fuels, through the construction, to exploitation waste management, is considered in the analysis. In 2014, the Intergovernmental Panel on Climate Change harmonized the equivalent results of carbon dioxide emissions (CO_2_e) from the main sources of electricity used in the whole world (IPCC 2014). The advancements in the efficiency of new generation wind and nuclear power plants, when it comes to the reduction of CO_2_e until 2017 (Pérez de Arce et al. 2016), is not considered in the analysis. The external costs of atmospheric emissions of classical pollutants, in addition to CO_2_e, are summed up in Table 8.

**Table 8:** External costs of atmospheric emissions of classical pollutants in 2010 and 2020, €/t. Source: CASES (2008)

EU-27 average	2010	2020
Human health		
NH3	9482	5837
NMVOC	584	238
NOx	5591	6620
PPMco	1325	1381
PPM25	24,410	24,191
SO2	6070	6673
Loss of biodiversity		
NH3	3266	3295
NMVOC	-67	-48
NOx	903	868
SO2	177	192
Crops: regional: crops N deposition & crops O3		
NH3	-183	-183
NMVOC	189	103
NOx	328	435
SO2	-27	-41
Crops: SO2		
SO2	-13	-13
Materials: SO2 & NOx		
NOx	71	71
SO2	259	259

When analyzing the costs of electricity generation by using different technologies, it should be emphasized that renewable energy can fully compete with fossil fuel technologies. Wind and solar energy have external costs lower than fossil technologies and comparable with nuclear energy. The latter is a low-emission one, but carries the risk related to the release of nuclear radionuclides. In the assessment of quantifiable external costs, the costs of greenhouse gas emissions play a key role. The literature on the subject defines the order of magnitude of the social cost of coal values in different ways, which significantly affects the assessment of the external costs of fossil fuel technologies. Exploitation, transport, and conversion of energy sources from fossil fuels and biomass invariably lead to the release of various forms of pollution into the environment. These pollutants can affect the quality of air, water, and soil and they can have a negative impact on human health. Unlike greenhouse gases, which are quickly mixed in the Earth’s atmosphere, these emissions can have different effects and therefore different costs, depending on the location and characteristics of the source of pollution. As a third tangible type of external costs, the costs of landscape distortion and noise interference are important only for the wind energy which is produced on land and sea.

The cost-benefit analysis is the primary application area of the research results applicability in terms of estimation of the external costs in the production of electricity. It is usually carried out with reference to the so-called public goods, but its scope reaches the issues of environmental protection as well. The cost-benefit analysis is utilized to determine the environmental goals and to select the policy that will help to reduce the negative impact of human activity on the environment.

## Case studies: environmental impact of selected technologies

### Case study: Czorsztyn hydroelectric power station

Pieniny is a mountain range located near the border between Poland and Slovakia. It is a region unique on a European scale, with numerous unique ecosystems featuring epilithic xerothermic grasslands. They were formed due to the diversity of landform and topography, the specifics of bedrock, and the microclimate. The fauna and flora of Pieniny cannot be found in any other region of Poland (Witkowski 2003). These areas are also very precious for monitoring the environmental changes that are caused by the construction of large water reservoirs. The construction of the Czorsztyn-Sromowce dam, consisting of two large reservoirs, had been planned since at least the 1950s. Because of these plans, Pieniny was subjected to constant scientific monitoring of the microclimate, environmental characteristics, and the flora and fauna (Żukowski 1957). The reservoirs were filled in the period 1994–1996. Their capacities are 231.9 and 6.7 million m^3^, and their areas are 1,226 ha and 88 ha, respectively.

The case study, which includes the results of the aforementioned scientific monitoring, presents the changes that have occurred in the fauna of noctuids (Lepidoptera, Noctuidae) of xerothermic rock ecosystems in the Pieniny and that have resulted from the construction of the water reservoirs in Czorsztyn-Niedzica and Sromowce Wyżne. The analysis of those changes throughout about 40 years was carried out on the basis of historical research on Lepidoptera, including the noctuids of the Pieniny (SBJR and Zukowski 1965), as well as the results of contemporary research on the Pieniny noctuids (Nowacki 2010; Nowacki and Węsala 2008), conducted on 12 sites. Using comparative analysis, significant changes were found in the noctuid communities of rock xerothermic ecosystems. Out of 25 xerothermophile species occurring in the Pieniny in the mid-twentieth century, 8 species were not confirmed. The analysis of changes leads to conclusions that they had been caused to a great extent by the construction of the water reservoirs located at the foot of Pieniny. This can be clearly seen in the Czorsztyn castle site, currently situated in the immediate vicinity of the reservoir, where out of 19 characteristic species as many as 10 were not confirmed, while 1 species not observed in the previous research was reported. It should be stressed, however, that the lack of influence of the water reservoir system Czorsztyn-Niedzica and Sromowce Wyżne on the main pollinator insects: butterflies (Rophalocera), bumblebees, and cuckoo bees (Bombinii), was noticed (Adamski et al. 2010).

Other observations indicate some serious changes in weevil fauna in the area of the complex of the Water Reservoirs Czorsztyn-Niedzica and Sromowce Wyżne in a relatively short time and they show the influence of strong anthropogenic pressure (Knutelski et al. 2010). The changes in the studied entomofauna are the effect of abiotic and biotic transformations in the environment of that region. The range of these changes is large and it embraces species richness, species composition, biodiversity, abundance, and dominance structure of weevils. The most sensitive are some stenotopic species (thermophilous, “rare” and mountainous).

More holistic studies concerning the fauna in Pieniny were conducted by Knutelski (2010). The fauna of fish, amphibians, birds, mammals, invertebrates zoobenthos, molluscs, and terrestrial insects (weevils, owlet moths, and pollinating insects—butterflies and bumblebees) have been studied in the area of the artificial complex of Water Reservoirs Czorsztyn-Niedzica and Sromowce Wyżne in different research periods. Species exchange included all studied animal groups, and the transformation of the owlet moths, weevils, butterflies, birds, and fish fauna was the highest. The changes in other groups of animals are less visible. Spectacular changes in the abundance have been noted in almost all amphibian species and fish, in some bird and zoobenthos species, as well as weevil and thermophilous owlet moth species. In most of the groups, the abundance decreased although the specimen number of some bird, fish, weevil, and butterfly species increased. Both the water fauna and the water-continental and xerothermophilous fauna, especially that of biotopes neighboring with reservoirs, are the most sensitive to environmental disturbance (Augustyn 2010). However, these changes were also observed in some more distant environments, although they are smaller.

The analysis of the changes in particular sites allows to conclude that the construction of water reservoirs at the foot of Pieniny played a major role in causing those changes.

### Nuclear power

The Chernobyl and Fukushima disasters were, so far, the only accidents that have the highest INES level 7 rating (Bolsunovsky and Dementyev 2011; Steinhauser et al. 2014). Let us consider their impact on the environment.

#### Case study: Fukushima disaster

On 11th March 2011, a tsunami wave, caused by the earthquake of 9 magnitude, damaged the Fukushima Daiichi nuclear plant. As a result, several reactors started to melt down and radionuclides were released into the environment (Brumfiel and Cyranoski 2011). The radiation monitors at the Daiichi plant showed 400 millisieverts per hour, which exceeds the legal limit 400 times (Brumfiel and Cyranoski 2011), whereas in the sea in the immediate vicinity of the plant, the water monitors showed that the legal limit for radioisotopes in public water exceeded 7.5 million times. It was a consequence of the inflow of highly radioactive water to the ocean (Reardon 2011). According to various observations, estimations, and models, the total amount of radioactive isotopes released to the air were 105.9 ÷ 160 PBq^15^ (petabecquerel, i.e., 10^15^ Bq) for ^131^I (the superscript on the left denotes the mass number of the isotope), around 35.8 PBq for ^132^I, 11.6 ÷ 36.7 PBq for ^137^Cs, and around 15.3 Ebq (exabecquerel, i.e., 10^18^ Bq) for ^133^Xe. Similarly, it is estimated that 1 ÷ 42 PBq of ^137^Cs and 0.1 ÷ 22 PBq of ^90^Sr were released to the Pacific Ocean (Aliyu et al. 2015).

As a result, the impact of the disaster was global—the radioactive isotopes related to the Fukushima accident were detected in locations all over the world (Aliyu et al. 2015; Bolsunovsky and Dementyev 2011; Manolopoulou et al. 2011). It is too early to assess the long-term impact of the Fukushima accident on the environment. Nevertheless, both observations and models show that the ^137^Cs radioisotope is accumulated in marine food chains which is, among others, caused by the Fukushima accident (Alava and Gobas 2016; Reardon 2011). On the other hand, however, cesium radioisotope activity do not indicate a health concern for the consumption of seafood outside of Japan (Alava and Gobas 2016; Aliyu et al. 2015). Furthermore, marine organisms are reported to have great resistance to nuclear radiation, although a significant amount of radioisotopes can be accumulated in marine biomass (Reardon 2011).

The studies concerning the influence of the Fukushima Accident on terrestrial organisms near Fukushima showed various physiological, morphological, and developmental pathologies that had been caused by exposure to radioactivity. Morphological and genetic pathological changes in butterflies, hematological aberrations in monkeys, a decrease of the population of birds, butterflies, and cicadas as well as aberrant growth forms in trees were observed (Aliyu et al. 2015). The maximum number of human mortality is estimated to be 10,000 cases, whereas for cancer it is 1800 cases (Aliyu et al. 2015; Hoeve and Jacobson 2012). The main non-cancer mortality reason is connected with the aggravation of chronic diseases due to the disaster. A significant decrease of public support for the nuclear power industry can be observed (Kim et al. 2013). The necessity of creating an effective safety system, especially in the countries in which the nuclear power industry is developed, is stressed (Aoki and Rothwell 2013).

#### Case study: Chernobyl disaster

The Chernobyl nuclear accident took place in 1986. It was caused by an operating error which led to the destruction of the reactor and explosion. According to various observations and estimations, the total amount of the radioactive isotopes released to the air was 1200 ÷ 1700 PBq for ^131^I, 74 ÷ 98 PBq for ^137^Cs, and about 6.5 Ebq (exabecquerel, i.e., 10^18^ Bq) for ^133^Xe (Steinhauser et al. 2014).

The thirty-year time horizon allows scientists to monitor not only the direct contamination of the natural environment but also the genetic disorders in various organisms. Genetic effects were investigated in mice from 1986 to 1994 in the polluted regions (Pomerantseva et al. 1997). Embryo mortality increased only in the progeny caught in 1987 in the area with maximum contamination. The frequency of mice heterozygous for recessive lethal mutations was initially high, but it decreased with time after the accident. The genetic consequences of the radioactive contamination to agricultural crops were significant as well (Geraskin et al. 2003). It turned out that a chronic low dose can cause an inheritable destabilization of genetic structures occurring, in particular, as the increase in cytogenetic damage and karyotypic variability in the offspring of irradiated organisms. Pathological changes in DNA histograms in species of the fish collected within a 10-km radius of the Chernobyl Nuclear Power Plant were observed (Dallas et al. 1998). Changes in the blood of the children exposed to radiation from the Chernobyl accident were examined as well (Ben-Amotz et al. 1998). In total, there are 37 casualties directly caused by the explosion, and about 6000 people are estimated to have died as the consequence of the disaster (Marples 1996).

#### Case study: Diablo Canyon

There are also some long-term effects of nuclear power plant occurrence, mainly related to heat dissipation. The Diablo Canyon nuclear power plant in California is one such example. In 2018, it supplied 9*%* of California’s in-state electricity generation.^16^ It circulates 11.5 billion liters of seawater as a coolant. The impact is twofold. First of all, it sucks in fish larvae that die in the process. Secondly, warm water it releases back into the ocean creates an artificial ecosystem (Sneed 2013). Both processes are closely monitored but conclusions on the impact are not coherent. Killing fish and fish larvae is a fact. However, it is disputable if this amount disturbs the ecosystem at all. The fish death toll is far greater from California’s coastal fishing industry (Boisvert 2015). The disturbing nature of the artificial ecosystem caused by heated water is not that certain as well. The documented impact is mainly at the proximity of the exhaust pipes, since surfacing warmer water spreads at the surfaces and cools down rapidly. Building cooling towers is an alternative to using seawater. However, it would significantly affect the landscape and it might also have influence on the environment, regardless of the investment cost. On the other hand, Diablo Canyon strongly contributes to limiting carbon emissions. Its generation is comparable with all wind turbines and rooftop photovoltaics in California. Replacing it with an alternative generation is challenging because of its significant contribution to electricity generation. The level of energy production by nuclear power plants in California has remained stable over recent years, while the percentage of renewable energy is increasing. Accordingly, the percentage share of the nuclear power plant in carbon-free production in California was 18*%* in 2018.^17^

### Wind power

Wind turbines are a source of renewable energy with a relatively minor impact on the environment, in comparison with fossil fuel-based generation (Guezuraga et al. 2012). The reduced CO_2_ emission is the most obvious positive effect of wind power on the environment. Wind power also contributes to the reduction of water consumption—it features one of the lowest liter-to-kWh ratios among all generation technologies (Gipe 1995).

It is assumed that 1 MW of wind power requires an area of circa 1 ha, though this value varies greatly according to the ease of access for construction and operation, technical parameters, and environmental specificity (Tegen 2015). It also has to be noted that the land required for turbine foundations and access roads is a small fraction of the overall area, allowing other (e.g., agricultural) utilization of the space.

Wind turbines generate constant low-intensity noise and vibration which consists of air-borne and structure-borne components. Theoretically, these can generate stress which, in turn, may affect health. However, at the distance of several hundred meters, the noise level drops to acceptable levels and becomes inaudible within circa 1.5–1.9 km (Gipe 1995; Dai et al. 2015). Regulations usually define the minimum distance between wind farms and residential buildings. It often results from the function of turbine height, although specific rules vary locally (Nieuwenhuizen and Köhl 2015). The standards for assessment of wind turbine installations (including measurement of environment-related parameters) are defined by the IEC 61400 standard. The effects of wind turbine noise on livestock were assessed in Mikolajczak et al. (2013), in which it was proved that the levels of cortisol (stress hormone) in geese bred within 50 m from a wind turbine was noticeably higher than in those bred 500 m away. This is in line with the aforementioned guidelines for wind farm siting.

The presence of wind farms may slightly affect the local microclimate. The study conducted in Scotland showed a slight increase in temperature and humidity during some periods of the day (Armstrong et al. 2016). Although this impact is insignificant in comparison with other types of electricity generation (especially non-renewable), it slightly affects the overall carbon balance of wind energy.

The environmental threat for birds and bats is one of the most disputed. It occurs mainly as a result of disturbance, loss of habitat, and the risk of collision. The last one is recognized as the most significant. The data varies greatly among sources, mostly due to the differences in methodologies and characteristics of the plants assessed. The general consensus is that the nature of the influence is complex, and although correctly positioned wind farms do not pose an excessive risk to birds (in comparison with other threats), the situation still needs to be monitored. The Royal Society for the Protection of Birds calls for more strategic planning and analysis of bird migration routes when new wind power facilities are developed (Royal Society for the Protection of Birds 2017). The turbine height is the most significant factor that determines the collision risk (Loss et al. 2013). A much-discussed study suggests some methodological errors in many attempts to estimate avian mortality due to various types of power generation and estimates that the fatalities due to wind power (0.269 fatalities/GWh) are lower than those due to nuclear power (0.416 fatalities/GWh) and significantly lower than those related to fossil fuel-based plants (5.18 fatalities/GWh) (Benjamin 2009).

One study estimates that 51 GW of wind power killed 880,000 bats and 573,000 birds, including 83,000 raptors, in 2012 in the USA. Thus, the 8 GW of wind turbines needed to replace Diablo Canyon’s output would likely kill hundreds of thousands of bats and birds each year.^18^ California’s Ivanpah concentrating solar plant has won notoriety for roasting birds in mid-air with focused sunlight from its mirrors.

The Nysted offshore wind farm, also known as Rødland I, is situated close to the Rødland bank in Denmark. It was built in 2003 and it consists of 72 turbines of a total capacity equal to 166 MW. In 2010, an over 200-MW extension, known as Rødland II, was installed. Since the plant installation, the natural environment close to the wind farm has been monitored in the context of the influence of the farm on nature. The population of the harbor porpoise (*Phocoena phocoena*) was monitored using acoustic detectors (Teilmann and Carstensen 2012). Immediately after the foundation of the turbines, the population of porpoises in the vicinity of the wind farm decreased rapidly to 11*%* of the initial state. Gradual increasing was then observed, and in 2012 the population inside Nysted offshore farm reached 29*%* of the baseline level. It should be stressed, however, that the decrease of porpoise habituation was not observed in Rødland II based, similarly to the Nysted farm, on gravity foundations and, as a consequence, there was no pile driving during placement (Hammar et al. 2016). Furthermore, some monitoring results from another offshore wind farms in the Baltic and North Sea showed that porpoise inhabitation returned to normal levels after the construction works (see (Hammar et al. 2016) and references given there) or even increased in comparison with the level before placement (Scheidat et al. 2011). Therefore, it is supposed that the declination observed in Nysted was ostensible. It can be an effect of natural fluctuations. The observed effect may have been caused by a short observation period of porpoises activity in Nysted before the farm construction. In order to study the influence of construction works and the operating of turbines of the Nysted plant on the behavior of harbor seals (*Phoca vitulina*), an observation of seal haul-out behavior was performed. The monitoring was conducted during two preconstruction periods (total of 8 months), the construction period (7 months) and the period of operation (one year) (Edrén et al. 2010). In general, no long-term effects on the haul-out behavior of seals were found in comparison with neighboring haul-out sites. Only a short-term decrease in the number of seals, caused by sheet pile driving, was registered. The seal population increased in 2004–2005, which was probably caused by creating an artificial reef at the base of the wind turbines. The reefs attracted fish whose increased presence was attractive for seals (Teilmann et al. 2006). The problem of bird collisions with wind turbines is the best-recognized issue related to environmental protection in the context of wind energy. Possibilities of monitoring of bird mortality caused by collisions with wind turbines in offshore wind farms are limited due to the problems of detecting these collisions at sea. Nevertheless, radar monitoring at Nysted shows that birds quickly learn to avoid the turbine cluster, during a day at a distance of about 3 km and at night about 1 km away. It was shown that the number of waterbirds entering the Nysted wind farm area decreased by a factor 4.5 for preconstruction to initial operation (Desholm 2006). For instance, the density of long-tailed ducks (*Clangula hyemalis*) on the farm decreased after the farm placement, but the total number of ducks in the entire area did not change (Petersen et al. 2011). Furthermore, at Nysted, where the turbines are 480 m apart, some birds are able to fly safely among them (Desholm 2009; Drewitt and Langston 2006). The observations indicate that approximately only 0.02*%* of the birds collide with turbines (Snyder and Kaiser 2009). The aforementioned artificial reef effect is associated with the settlement of various organisms on the bases of turbines immersed in water. Blue mussels (*Mytilus edulis*) mounted on the upper regions of the underwater part of a turbine pole achieve 7–18 times greater mass than the ones settled close to the seabed because of rich nutrients supply in these layers of the sea Maar et al. (2009). The spots of high biomass of mussels create centers of biological activity in which the ecological dynamics are stimulated, among others by intensive ingestion of microplankton and copepods.

### Solar power

There were a few solar power plant accidents in the past. In 1990, there was a series of explosions at the Harper Lake CSP, California. The plant was generating 80 MW at that time. As a result of an equipment malfunction, there was a fire that consumed about 69,000 liters of synthetic oil (Stammer and Harris 1990). In 2016, a mirror misalignment caused a fire at the Ivanpah Solar Electric Generating System in the Mojave Desert, California. It was confirmed that the misalignment happened because the mirrors did not track the sun properly. The Ivanpah generates 392 MW, which makes it one of the largest CSP plants in the world. The accident resulted in the melting of some cabling and pipes. Since the plant is water-based, no toxic substances were released to the atmosphere. On the other hand, the plant is a kind of threat for birds. As it has been estimated by BrightSource Energy, one of the owners, about 1000 birds are roasted every year. Other sources claim these numbers to be much higher, reaching 28,000 (Anthony 2014). Such reports of significant numbers of birds killed by concentrated sunlight seem to contain misinformation and to be exaggerated. Avian mortality studies at concentrating solar power plants (Ho 2016) indicate that it is from 0.7 to 3.5 fatalities per GWh. It is less than the volume reported for fossil fuel plants. The impact on the local and migratory bird populations was determined as low.

The accidents with CSP plants seem to be rare. Even if they do occur, there is only a minor environmental impact. CSP saw 100 MW of capacity come online in 2017, bringing global capacity to about 4.9 GW. Several projects that were due to enter operation during the year were delayed until 2018 and later. Although global capacity increased by just over 2% in 2017, the CSP industry was active, with a pipeline of about 2 GW of projects under construction around the world, particularly in China, the Middle East, and North Africa (MENA) region.

### Coal-based power

The problem of emission reduction for fossil fuel power plants is well covered. In 2006, a zero-emission fossil fuel plant concept was introduced in the EU (Platform 2006). Thus, the emission constantly decreases, while the efficiency increases, both for retrofit and newly built plants. Let us use the Krakow Power Plant as an example. It is rated at 460 MW of electrical power and 1547 MW of heat. It was commissioned in 1970 and started to operate at full capacity in 1986. Due to constant modernization, it is expected to decrease its PM emission by a factor of 17, comparing 2007 and 2016^19^. In 2016, there was a 120 million € investment targeted at decreasing sulfur oxide emissions from 5 to 7 times and nitrogen oxides emissions from 2 to 3 times, simultaneously decreasing PM emission by a factor of three (PC 2012).

Nevertheless, there is also some indirect impact on the environment caused by coal mining operations. In Poland, Upper Silesia is a region with intensive coal mining since the end of the eighteenth century. Therefore, a lot of coal-mine heaps which differ in terms of their age, area, the character of the surroundings, the way in which they were created, and the occurrence of thermal activity, are situated there. Moreover, various characteristics of local habitats can be observed. The habitats can differ in factors such as moisture, salinity, coarseness, compactness, and the character of vegetation in the adjacent neighborhood. Therefore, Upper Silesia is a good region for extended studies concerning the restoration of post-mining lands in various aspects—(Stefanowicz et al. 2015; Woźniak et al. 2015) and (Woźniak 2010) can be put as examples of such studies. The main result is that richness and diversity of plant species on coal-mine heaps rise with time. Moreover, there is no significant difference with regard to the site area. However, thermal activity, coarseness, and moisture are associated with differences in vegetation. The factors that describe the quality of plant vegetation such as the plant height, area of leaves, root system, seed mass frequency, and time of germination are correlated positively with the age of heap (Woźniak 2010). This means that vegetation transforms hostile post-mining regions gradually into milieus more and more biologically friendly.

## Concluding remarks

To sum up the presented review, let us specify the following remarks:

1. Climate change and environmental pollution make it necessary to reduce the use of non-renewable resources.
2. Breakdown effects of the power stations based on renewable energy, primarily of wind and solar farms, are limited to the station, whereas breakdown of power stations based on non-renewable resources, primarily nuclear plants, can have global consequences.
3. It is not true that all types of renewable energy are clean in terms of pollutants and emissions. Biomass combustion and dams emit harmful gases whereas mass cultures of energetic plants degrade the natural environment. Solar energy, wind energy, and the energy acquired from the kinetic energy of water and thermal energy of natural geysers are the only clean ones.
4. Solar and wind energy are troublesome and expensive but they are becoming more and more cost-competitive. Moreover, if the influence of non-renewable energy on the environment, including both human health and high risk connected with breakdowns, is taken into account, it turns out, that on the whole, clean energy is profitable.
5. Problems with the utilization of the worn-out materials used for clean energy production should be monitored carefully and studied intensively.

Health and environmental impacts have to be taken into consideration when planning electricity generation. Combustion of fossil fuels has a significant impact on human health because two-fifths of the human population is exposed to the air pollution caused by it (Smith et al. 2013). It should be stressed that the problem of external costs should be studied carefully because it depends strongly on the fact whether the studied region is already developed or still developing. The differences in the amount of external costs are determined by such specific factors as population density, meteorological conditions, and average emissions caused by the existing electricity grid. Therefore, the results of analyzes differ in the case of developed and developing countries. Health costs and the greenhouse effect clearly dominate over other effects, contributing about 98%. When transferring financial valuations regarding the value of life and health between developing and developed countries, there are difficulties associated mainly with the differences in the level of income (difference in WTP value), in the age distribution of population, and mortality rates. In recent years, fast progress in reducing environmental pollution is observed. In many developing countries, however, electric energy production is based mainly on coal or wood combustion. Due to the fuel structure of the power industry, the current large differences in the emission ratios between countries will not change significantly in the next years. It should also be stressed that the size of health effects is correlated first of all with the density of the population in the exposed areas. Damage calculated per ton of emitted pollutant depends, to a great extent, on the location and physical characteristics of the emission source affecting the spread of pollutants. Long-term greenhouse gases are the only exception. Uniform mixing can be assumed for them in the whole atmosphere and consequently, there is no dependence of damage on the location of the emission source.

The combustion pollution effect, including the pollutants generated by the power industry, is global, which has been confirmed by empirical tests and theoretical models (van Zelm et al. 2016). It needs to be stressed that harvesting solar energy does not generate any byproducts, and therefore it is a truly clean energy source. Contemporary research in this domain focuses on the improvement of harvesting efficiency and new material applications (Schwarzbözl et al. 2006; Fend et al. 2013; Polman et al. 2016).

To sum up, the findings show that it is profitable to invest in truly clean renewable energy, i.e., in wind power, solar, and geysers that have a minimal negative impact on the natural environment. Similarly, potential impact due to a malfunction is also minimal.

## Footnotes

1 Acronym of “External Costs of Energy”: European Commission. 1995. Externalities of Energy: ExternE Project. For the Directorate General XII. Prepared by Metroeconomica, IER, Eyre Energy-Environment, ETSU, Ecole des Mines. Luxembourg: Office for Official Publications of the European Communities.

2 RFF/ORNL pertains to research conducted jointly by Resources for the Future and the Oak Ridge National Laboratory.

3 http://ecosenseweb.ier.uni-stuttgart.de/

4 CORe INventory of AIR emissions. CORINAIR is a project performed since 1995 by the European Topic Centre on Air Emissions under contract to the European Environment Agency. The aim is to collect, maintain, manage, and publish information on emissions into the air, by means of an inventory and database system for European air emission data. This concerns air emissions from all sources relevant to the environmental problems of climate change, acidification, eutrophication, tropospheric ozone, air quality, and dispersion of hazardous substances. The geographical scope of the current CORINAIR project includes the 15 EU Member States and a few other European countries. http://etc-ae.eionet.eu.int/etc-ae/index.htm

5 New Elements for the Assessment of External Costs from Energy Technologie. Project financed by the European Union, DG Research, Technological Development and Demonstration (RTD), http://www.ier.uni-stuttgart.de/forschung/projektwebsites/newext/

6 http://www.externe.info/externe_d7/?q=node/59

7 New Energy Externalities Developments for Sustainability. Project no: 502687. http://www.needs-project.org/ The goal of the NEEDS was the estimation of the total costs and benefits (direct and external) of energy policy and future energy systems, both on the level of the EU and the individual countries.

8 CASES (Cost Assessment of Sustainable Energy System). D 3.2 deliverable. Report on the monetary valuation of energy-related impacts on land use changes, acidification, eutrophication, visual intrusion, and climate change. EC; 2006.

9 The current version of EcoSenseWeb determines the impact of the emissions of “classic” pollutants as well as of some of the most important heavy metals in different spacial scales. It also includes the assessment of the harm caused by greenhouse gases emission. http://ecosenseweb.ier.uni-stuttgart.de/

10 MARKAL was developed within the framework of the international Energy Technology Systems Analysis Programme (ETSAP) of the International Energy Agency. The basic elements of the model are specific types of technologies for controlling energy and emission. Each of them is represented quantitatively by a set of efficiency and cost parameters. A menu of both the existing and future technologies is introduced into the model. The supply and the demand sides are integrated, thanks to which either side reacts to changes in the other. The model chooses the combination of technologies which minimizes the costs of a energy system. http://iea-etsap.org/index.php/etsap-tools/model-generators/markal

11 The phrase “internalization of external costs” denotes the rule where all costs connected with pollution emission should be included in the production costs of an enterprise which generates pollution.

12 A Scenario Generator is a simulation tool which generates a virtual population which reflects the demography of a real population and conditions witch are randomly distributed according to the known data on the frequency of occurrence.

13 Model for Energy Supply Strategy Alternatives and their General Environmental Impact (MESSAGE), International Institute for Applied Systems Analysis 26th April 2009, http://www.iiasa.ac.at/Research/ENE/model/message.html

14 CO2DB is a database with detail information on the technologies for the reduction of carbon dioxide emission. The database holds around 3000 technologies now. This includes detailed technical, economic, and environmental specifications as well as information on innovation and commercialization.

15 The becquerel (Bq) is a unit of radioactivity in the International System of Units (SI). The radioactivity of a radioactive sample is equal to 1 Bq if one nucleus decays per one second.

16 California Energy Commision, CEC-1304 Power Plant Owners Reporting Form, 2019

17 California Energy Commision, CEC-1304 Power Plant Owners Reporting Form, 2019

18 the Breakthrough Institute, https://thebreakthrough.org/issues/energy/diablo-canyon-nuclear-power-shutdown-risk

19 Krakow Naszemiasto, http://krakow.naszemiasto.pl/artykul/elektrocieplownia-w-legu-bedzie-bardziej-ekologiczna,2741024,art,t,id,tm.html
